# Supplementary material for: MetaRibo-Seq measures translation in microbiomes
Source: Nat Commun. 2020 Jun 29;11:3268. doi: 10.1038/s41467-020-17081-z (PMC7324362; doi:10.1038/s41467-020-17081-z)
Supplement: Supplementary file 10 — Supplementary Data 7 [file 41467_2020_17081_MOESM10_ESM.zip › File2/Confidence_High_Taxonomy/182902_out.krona.html]

Javascript must be enabled to view this page.

members
magnitude
magnitudeUnassigned
count
unassigned
taxon
rank

182902\_out

6

2
6
superkingdom

976
phylum
6

200643
class
6

6
order
171549

171552
6
family

6
genus
838

165179

SRS016541\_contig\_number\_11858SRS017307\_contig\_number\_22794SRS049959\_contig\_number\_35003SRS1041031\_contig\_number\_30104SRS1041140\_contig\_number\_19358SRS144537\_contig\_number\_44772
species
6
